# An in‐house protocol for improved flood field calibration of TrueBeam FFF cine imaging

**DOI:** 10.1002/acm2.12023

**Published:** 2017-01-19

**Authors:** Austin M. Faught, Fang‐Fang Yin, Justus Adamson

**Affiliations:** ^1^ Department of Radiation Oncology Duke University Medical Center Durham NC 27710 USA; ^2^ Department of Radiation Oncology University of Colorado School of Medicine Aurora CO 80045 USA

**Keywords:** calibration protocol, flattening filter free (FFF), MV portal imaging, stereotactic body radiation therapy (SBRT)

## Abstract

**Purpose:**

TrueBeams equipped with the 40 × 30 cm^2^ Electronic Portal Imaging Devices (EPIDs) are prone to image saturation at the image center when used with flattening filter free (FFF) photon energies. While cine imaging during treatment may not saturate because the beam is attenuated by the patient, the flood field calibration is affected when the standard calibration procedure is followed. Here, we describe the hardware and protocol to achieve improved image quality for this model of TrueBeam EPID.

**Materials & methods:**

A stainless steel filter of uniform thickness was designed to have sufficient attenuation to avoid panel saturation. The cine imaging flood field calibration was acquired with the filter in place for the FFF energies under the standard calibration geometry (SID = 150 cm). Image quality during MV cine was assessed with & without the modified flood field calibration using a low contrast resolution phantom and an anthropomorphic phantom.

**Results:**

When the flood field is acquired without the filter in place, a pixel gain artifact is clearly present in the image center which may be mis‐attributed to panel saturation in the subject image. At the image center, the artifact obscured all low contrast inserts and was also visible on the anthropomorphic phantom. Using the filter for flood field calibration eliminates the artifact.

**Conclusion:**

TrueBeams equipped with the 40 × 30 cm^2^ IDU can utilize a modified flood field calibration procedure for FFF photon energies that improves image quality for cine MV imaging.

## 1 Introduction

Lung and liver SBRT involve high‐dose treatments in few fractions, and commonly employ flattening filter free (FFF) photon energies.^1^, ^2^ Localization accuracy is essential for these treatments,^3^ with various motion management strategies being employed. Lung tumors or implanted fiducials in the liver^4^ are often visible on cine MV imaging using the electronic portal imaging device.^5^ When possible, cine imaging using the MV beam during 3D conformal SBRT is one of the best methods to verify target localization; this is because the tumor is visualized during the treatment in the beam's eye view. However, for Varian TrueBeam EPIDs that utilize the 40 cm × 30 cm Image Detection Unit (IDU), we have found that the current vendor procedures result in a saturated flood field at the image center, leading to compromised image quality for cine imaging using an FFF beam. We describe the required protocol and hardware to apply a correct flood field and achieve high quality imaging for this imaging mode.

## 2 Methods

All tests were performed on a TrueBeam linear accelerator (Varian Medical Systems, Palo Alto, CA, USA) running TrueBeam MR 2.5, with the 40 cm × 30 cm IDU. We acquired flood field images according to the vendor specifications, 150 cm source to imager distance (SID), for both 6 MV and 10 MV‐FFF energies at all clinical dose rates. Upon examination of the flood field images, we found that saturation occurred for the 10 MV‐FFF beam at the two highest dose rates (2000 MU/min, and 2400 MU/min). Stainless steel (nominal density = 8.03 g/cm^3^) plates measuring 25.4 × 25.4 cm^2^ and uniform 1.27 cm thickness (tolerance = ± 0.14 cm) were placed on an accessory tray mounted to the gantry head; the tray was open in the center so that only the plates were attenuating the beam. With the plates and mount in place, a megavoltage portal image utilizing the 10 MV‐FFF energy at the highest allowable dose rate (2400 MU/min) was acquired. The profile of the image acquisition was measured and checked for saturated signal at the center of the profile where the dose rate is highest. Additional plates were added until the measured profile was no longer saturated in the central region.

**Figure 1:**
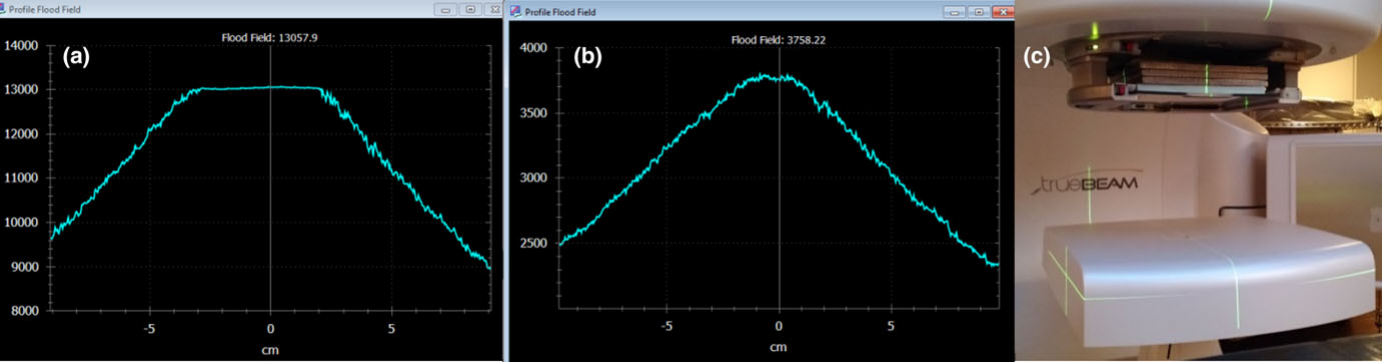
(a) Profile of a flood field image using the TrueBeam automated procedure for 10MV‐FFF photons (2000 MU/min dose rate) is shown. The flat intensity signal at the center of the profile is from saturation of the detector unit. (b) A profile of a flood field image using 3.8 cm uniform stainless steel filter to attenuate the beam shows no effects of detector unit saturation. (c) The filter apparatus rests on an accessory tray mounted to the gantry head.

Once the number of plates required to reduce the dose rate to allow for a non‐saturated image had been determined, calibration of the IDU was performed according to the manufacturer recommendations with the steel plates in place. Shown in Fig. 1 are measured profiles during the calibration procedure with and without the plates in place.

To test the new calibration, MV images of a low contrast quality assurance phantom and an anthropomorphic pelvis phantom were acquired a dose rate of 2000 MU/min using the flood field calibration file with the filter and without. For the comparison using the low contrast quality assurance phantom, the filter was placed in the beam's path to reduce the dose rate at the detector and allow for a readable image when using both calibration files. For the anthropomorphic pelvis phantom, the phantom attenuated the beam enough that the filter was not needed for either image acquisition. These images were then compared to assess differences in image quality when using the filter for flood field acquisition during the calibration procedure.

## 3 Results and discussion

**Figure 2:**
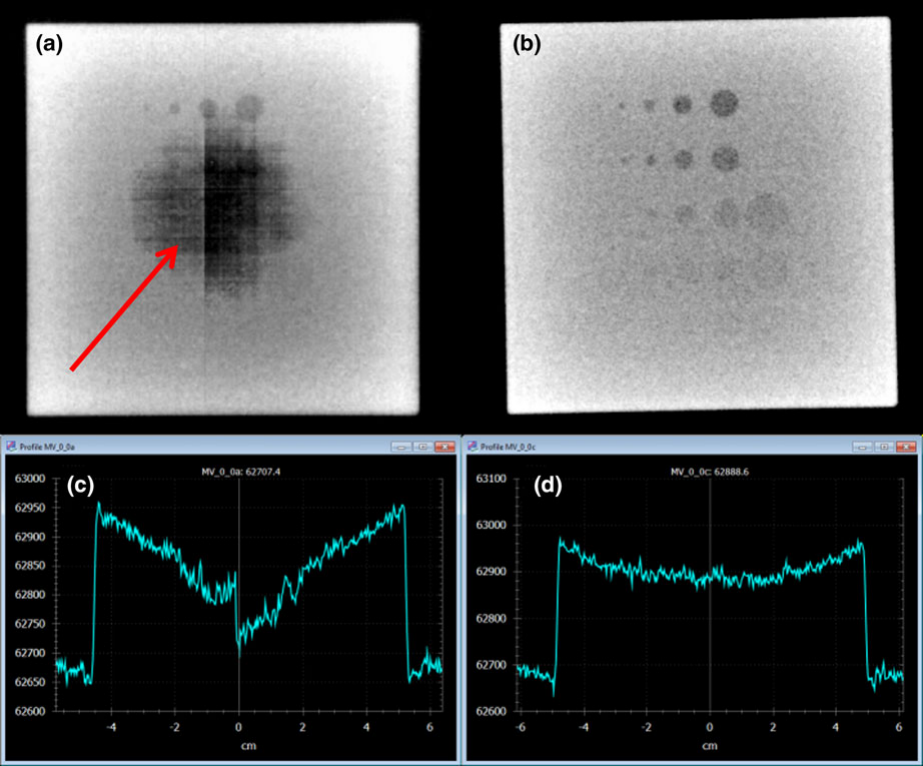
A 10MV‐FFF (2000MU/min) MV cine image of Vegas low contrast phantom acquired with flood field specified by Varian procedures (a) and a flood field with filter (b). During acquisition of (a) and (b) with the contrast phantom, the filter was used to attenuate the beam and avoid saturation. The artifact in (a) (indicated by arrow) is due to the saturation of the flood field. Images (c) and (d) are profiles across images (a) and (b), and demonstrate that the artifact in (a) is due to the flood field, rather than a saturation of image (a).

**Figure 3:**
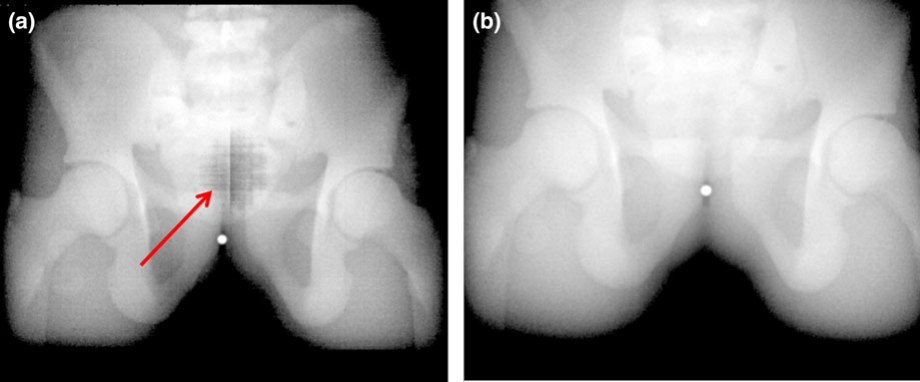
A 10MV‐FFF (2000MU/min) MV cine image of an anthropomorphic pelvis phantom was acquired with no filter in place for flood field calibrations with (b) and without (a) the filter. The artifact in (a) (indicated by red arrow) is due to the saturation of the flood field when acquired without the filter in place.

Three plates (total 3.8 cm thickness) were needed to avoid saturation for 10 MV‐FFF at 2400 MU/min dose rate. Observable improvements in image quality were noted for both the quality assurance phantom (Fig. 2) and the anthropomorphic pelvis phantom (Fig. 3) after the imager flood field calibration was performed with the plates in place. By removing the effects of saturation during the flood field acquisition, an accurate map of pixel by pixel sensitivity of the detector unit was acquired which eliminated the saturation artifact at the center of the two phantoms. Even though the phantoms attenuated the primary beam enough that the detector wasn't saturated at the delivered dose rate, the saturation of the flood field acquisition would carry‐over to the phantom images by way of an incorrect gain map. In images acquired for 10 MV‐FFF beams at 2000 MU/min, the application of an incorrect gain map was observed as an artifact of approximately 3 cm in diameter at the center of the image.

While moving the IDU further from the gantry head would be an equally effective way to reduce the dose rate at the measurement point, currently the automation of the calibration procedure only allows for the imager to be placed at an SID of 150 cm. Until the software allows for imager calibration at different SID values, inclusion of a uniform attenuation filter during the flood field acquisition of the calibration procedure may be necessary for facilities that acquire cine images with flattening filter free beams with the 40 cm × 30 cm IDU.

## Conflict of Interest

This research was not supported by extramural funding. A patent application has been submitted regarding the physical filter. Fang‐Fang Yin reports research and licensing agreements with Varian Medical Systems, not related to this study. Justus Adamson reports a consulting arrangement with Immunolight LLC and ownership with ClearSight Radiotherapy Products, both of which are unrelated to this study. Austin Faught has nothing to disclose.
